# The Metabolic Diversity of Different *Salsola* Species Valorized Through Untargeted Metabolomics and In Vitro Bioassays: The Importance of Phenolic Constituents

**DOI:** 10.3390/plants15020199

**Published:** 2026-01-08

**Authors:** Hajar Salehi, Marco Armando De Gregorio, Gokhan Zengin, Sakina Yagi, Gunes Ak, Enver Saka, Fevzi Elbasan, Evren Yildiztugay, Leilei Zhang, Stefano Dall’Acqua, Luigi Lucini

**Affiliations:** 1 Department for Sustainable Food Process, Università Cattolica del Sacro Cuore, 20123 Piacenza, Italy; shajar.salehi@unicatt.it (H.S.); marcoarmando.degregorio@unicatt.it (M.A.D.G.); leilei.zhang@unicatt.it (L.Z.); luigi.lucini@unicatt.it (L.L.); 2 Department of Biology, Science Faculty, Selcuk University, Konya 42130, Turkey; gokhanzengin@selcuk.edu.tr (G.Z.); akguneselcuk@gmail.com (G.A.); enverss2016@gmail.com (E.S.); 3 Department of Botany, Faculty of Science, University of Khartoum, Khartoum 13314, Sudan; sakinayagi@gmail.com; 4 Department of Biotechnology, Science Faculty, Selcuk University, Konya 42130, Turkey; fevzi.elba@gmail.com (F.E.); eytugay@gmail.com (E.Y.); 5 Department of Pharmaceutical and Pharmacological Sciences, University of Padova, Via Marzolo 5, 35131 Padova, Italy

**Keywords:** functional phytochemistry, metabolomic fingerprinting, antioxidants, chemotaxonomy

## Abstract

Five *Salsola* species have been studied as sources of bioactive compounds using a comprehensive, untargeted metabolomic and bioactivity assessment. Plant material was extracted using ethyl acetate (EA), water, and methanol (MeOH). *S. ruthenica* exhibited the highest total phenolic content (46.04 mg GAE/g, MeOH extract) and antioxidant capacity (DPPH: 47.21 mg TE/g; ABTS: 97.40 mg TE/g; CUPRAC: 141.38 mg TE/g; FRAP: 80.30 mg TE/g). Extracts of *S. stenoptera* and *S. ruthenica* showed potent cholinesterase inhibition, while *S. crassa* was notably active against tyrosinase. A total of 265 metabolites were annotated, revealing strong solvent- and species-specific differences in phenolic composition, as confirmed by AMOPLS analysis. Flavanols, anthocyanins, and lignans emerged as the major chemotaxonomic markers, based on PCA, contributing the most to the total variance. Strong correlations were observed between TPC and CUPRAC (r = 0.93) and between flavanols and DPPH (r = 0.70), suggesting functional relevance of these compounds in redox activity, confirming the importance of different classes of phenolic constituents. VIP markers also revealed species- and solvent-specific enrichments of metabolites. Regularized canonical correlation analysis (rCCA) further linked specific metabolites, namely Quercetin 3-O-glucosyl-xyloside and 6″-O-Acetylgenistin, the flavanone sakuranetin, the lignans Secoisolariciresinol, Anhydro-secoisolariciresinol, and Medioresinol, and p-Coumaric acid ethyl ester, with antioxidant functions. These findings underscore the pharmacological potential of *Salsola* species and highlight the importance of valorizing metabolic diversity in the search for new sources of health-promoting natural compounds. Furthermore, the work shows the need for a tailored solvent selection in bioactivity-guided phytochemical research.

## 1. Introduction

Across the arid and saline landscapes, *Salsola* species have carved out a unique ecological niche. These salt-tolerant plants (halophytic) are keystone species in the fragile desert ecosystem and bear increasing relevance in agriculture, pharmacology, and environmental management. *Salsola* belongs to the Amaranthaceae family and is considered one of its largest genera, though its exact taxonomic boundaries remain unsettled [1,2]. The genus *Salsola* L. comprises approximately 150 species, including annual shrubs, subshrubs, small trees, and perennial herbs, which are distributed across Eurasia, the Middle East, North Africa, and Australia. In Turkey, the genus *Salsola* is represented by 18 species and 23 taxa [3]. Despite their economic importance, most species have been poorly studied.

Beyond their ecological resilience, marked by drought tolerance, salt adaptability, and prolific seed production, *Salsola* plants are attracting growing scientific attention for their biochemical richness [4]. Previous investigations have identified a broad spectrum of bioactive compounds in *Salsola* species such as *S. kali* and *S. cyclophylla*, including flavonoids, phenolic acids, alkaloids, saponins, triterpenoids, sterols, volatile oils, lignans, coumarins, and glycosides [4,5]. In most species of the *Salsola* genus, the phytochemical profile is dominated by flavonoids, phenolic compounds, and phenolic acids, bioactive constituents that are well-known for their roles in plant defense and for their potent antioxidant, anti-inflammatory, and antimicrobial properties [6]. These constituents are associated with a wide array of pharmacological properties. Extracts from various *Salsola* species have demonstrated therapeutic effects such as analgesic, anti-inflammatory, antimicrobial (antiviral and antibacterial), anticancer, hepatoprotective, and cardioprotective activities [7,8]. Several species are also used in traditional medicine systems in different regions to treat hypertension (*S. kali*), skin diseases *(S. somalensis*), bone fractures (*S. laricifolia*), and to expel tapeworms (*S. kali*) and intestinal parasites *(S. kali*) [2,9,10]. Other species, such as *S. soda* and *S. kali*, also have industrial applications in soap production, cotton bleaching, and as sources of sodium carbonate.

Based on the literature information, the phytochemical composition of *Salsola* species is far from uniform. It is shaped not only by genetic diversity, plant developmental stage, and habitat-specific factors but also by the methods employed in their study [4,5,11]. Solvent-based extraction remains a cornerstone of phytochemical research, with each solvent selectively enriching for different classes of metabolites. Polar solvents such as water, ethanol, and methanol are particularly effective in solubilizing phenolics, flavonoids, and other antioxidant-rich compounds [12,13]. Thus, employing a comparative extraction approach allows researchers to capture a complete metabolic profile and better understand how solvent choice influences plant extracts’ phytochemical yield and bioactivity. Given the widespread distribution, chemical richness, and traditional use of *Salsola* species, systematic phytochemical profiling, especially using advanced analytical tools, is essential for unlocking their full pharmacological potential. Despite its rich ethnobotanical background and wide distribution, comprehensive metabolomic data for *Salsola* species remain limited, especially in comparative solvent-based extractions. Few studies have systematically explored the metabolite variation among multiple *Salsola* species using high-resolution metabolomics [14,15].

In this study, we investigated the untargeted metabolomic profiles and antioxidant properties of three independent solvent extracts (aqueous, ethyl acetate, and methanolic) obtained from five *Salsola* species: *S. crassa*, *S. stenoptera*, *S. kali*, *S. ruthenica*, and *S. nitraria*. Untargeted metabolomic analysis was carried out using ultra-high-performance liquid chromatography coupled with electrospray ionization quadrupole time-of-flight mass spectrometry (UHPLC-ESI/QTOF-MS), enabling high-resolution identification of a wide range of plant metabolites. In addition, we evaluated non-enzymatic antioxidant activities to assess the potential of these extracts as natural sources of free radical scavengers. This integrative approach, combining advanced metabolomics with antioxidant screening, comprehensively assesses the *Salsola* species’ chemical and functional diversity. The findings provide valuable information regarding species-specific and solvent-dependent variations in metabolite composition and may support the future development of plant-based therapeutic or nutraceutical applications.

## 2. Results and Discussion

### 2.1. Total Phenolic and Flavonoid Content Influenced by Species and Extraction Variables

Quantification of total phenolic content (TPC) and total flavonoid content (TFC) across five *Salsola* species revealed significant variation depending on both species and extraction solvent (Table 1). The highest TPC was observed in the methanolic extract of *S. ruthenica* (46.04 mg GAE/g), followed by its ethyl acetate (EA) extract (27.94 mg GAE/g) and water extract (25.68 mg GAE/g). This indicates a broad solubility of phenolics in *S. ruthenica*, with methanol providing the most efficient recovery, consistent with its high polarity and established ability to solubilize a wide range of phenolics, including both glycosylated and aglycone forms [12]. In contrast, for the remaining species, the highest TPC was generally achieved with EA extracts: *S. stenoptera* (27.32 mg GAE/g), *S. kali* (25.19 mg GAE/g), *S. nitraria* (23.84 mg GAE/g), and *S. crassa* (21.78 mg GAE/g), indicating enrichment of moderately polar phenolics, such as lignans and certain flavonoid subclasses. Aqueous extracts showed the lowest phenolic recovery across most species, except for *S. ruthenica*, where water extraction remained relatively efficient.

Flavonoid content (TFC) followed a similar species-solvent trend. The methanolic extract of *S. kali* yielded the highest TFC (20.50 mg RE/g), consistent with previous reports on the high flavonoid yield from *Salsola* leaves in mid- to late growth stages [4]. *S. crassa* also showed elevated TFC in both MeOH and EA extracts, supporting its flavonoid-rich profile identified later via UHPLC-QTOF. Flavonoid levels in aqueous extracts were consistently low, reinforcing that nonpolar or mid-polar solvents are preferable for efficient recovery. These findings underscore the strong influence of both genetic and solvent factors on phenolic extraction. Solvent polarity plays a dominant role, but species-specific metabolic composition is equally critical. For instance, *S. ruthenica* and *S. kali* appear to exhibit elevated phenolic biosynthesis, possibly linked to environmental adaptation or evolutionary divergence within the *Salsola* genus [2,15].

While the TPC and TFC values provide a global estimate of total phenolics and flavonoids, untargeted metabolomics allows for the resolution of individual phenolic subclasses and their specific abundance across species-solvent combinations. Therefore, the next sections provide a deeper insight into the semi-quantitative composition and its relation to functional activity.

### 2.2. Antioxidant Activities Across Species and Extraction

The antioxidant potential of various extracts from five *Salsola* species was assessed using six complementary in vitro assays: DPPH, ABTS, CUPRAC, FRAP, metal chelating activity (MCA), and phosphomolybdenum total antioxidant capacity (PPM). The results, summarized in Table 2, reveal substantial species- and solvent-dependent variation in antioxidant properties. The methanolic extract of *S. ruthenica* consistently showed the highest activity in DPPH (47.21 mg TE/g), ABTS (97.40 mg TE/g), CUPRAC (141.38 mg TE/g), and FRAP (80.30 mg TE/g), reflecting its superior electron-donating and reducing capabilities. These results align with its high TPC values (Section 3.1), supporting the well-established relationship between phenolic content and antioxidant potential [16]. Methanol was the most effective extraction solvent overall, particularly for redox-active phenolics. Its broad polarity range facilitates recovery of compounds such as flavanols, phenolic acids, and glycosylated flavonoids, key contributors to antioxidant mechanisms [12,17]. Ethyl acetate, while less polar, also extracted potent antioxidants in some species, notably *S. kali* and *S. stenoptera*, indicating the presence of mid-polar compounds such as lignans or lipophilic phenolic derivatives [18]. Aqueous extracts generally exhibited lower radical scavenging and reducing activity, consistent with the limited solubility of many key antioxidant phenolics in water.

Metal chelation activity (MCA), which is critical for limiting Fenton-type reactions that generate hydroxyl radicals, ranged from 13.81 to 21.96 mg EDTAE/g. The aqueous extracts of all five species performed best, likely due to the presence of hydrophilic chelators. Additionally, the methanolic extracts of *S. nitraria* and *S. stenoptera* displayed significant chelation activity (*p* < 0.05). This result is consistent with other studies reporting strong chelating activity from aqueous-soluble phenolic fractions [19]. In the phosphomolybdenum (PPM) assay, the ethyl acetate (EA) extracts of *S. kali*, *S. nitraria*, and *S. ruthenica* exhibited the highest values (*p* < 0.05), indicating that the total electron transfer capacity may be enhanced by mid-polar constituents, such as lignans or lipophilic flavones, that are preferentially extracted by EA [20].

### 2.3. Enzyme Inhibitory Activities: Species-Specific and Solvent-Associated Bioactivity Patterns

Inhibiting key enzymes can help to address some global health problems. For example, inhibiting acetylcholinesterase (AChE) and butyrylcholinesterase (BChE) can increase acetylcholine levels in the synaptic gap, which can positively affect the cognitive function of Alzheimer’s patients [21]. Additionally, inhibiting amylase and glucosidase can help to control blood glucose levels in diabetic patients [22]. Tyrosinase is a key enzyme in melanin synthesis, and inhibiting it can control hyperpigmentation problems [23]. Therefore, effective, safe, and natural inhibitors are required for these problems.

In the current study, enzyme inhibition profiling revealed distinct species-specific and solvent-dependent trends in bioactivity against five target enzymes (AChE, BChE, tyrosinase, α-amylase, and α-glucosidase), highlighting the differential therapeutic potential of *Salsola* species (Table 3). The strongest AChE inhibitory activity was observed in the EA extract of *S. ruthenica* (2.41 mg GALAE/g), followed by EA and MeOH extracts of *S. stenoptera* and *S. nitraria*. These values suggest a high concentration of alkaloid-like or flavonoid-based inhibitors, which are known contributors to cholinesterase inhibition in *Salsola* and related Amaranthaceae species [24]. BChE inhibition followed a similar profile, with *S. ruthenica* and *S. crassa* showing notable activity, especially in EA extracts. Solvent type strongly influenced enzyme inhibition. Methanolic and EA extracts consistently outperformed aqueous extracts, aligning with their ability to recover moderately polar bioactive compounds. Water extracts showed minimal activity across all enzymes, likely due to the poor solubility of key inhibitors in aqueous media.

The most potent tyrosinase inhibition was recorded for *S. crassa* (EA extract, 56.33 mg KAE/g), followed closely by MeOH extracts of *S. ruthenica*, *S. stenoptera*, and *S. kali*. This pattern suggests a high content of flavonoids or hydroxycinnamic acid amides in these species, as previously identified in other *Salsola* taxa with strong melanogenesis inhibition potential [25]. In contrast, α-amylase and α-glucosidase inhibition was generally weaker across species and solvents. The highest α-amylase inhibition (0.55 mmol ACAE/g) was observed in the EA extract of *S. ruthenica*, while *S. kali* (MeOH extract) exhibited the strongest α-glucosidase inhibition (1.00 mmol ACAE/g). Although these values are moderate, they align with earlier findings of dual enzyme inhibition by *S. tragus*, *S. baryosma*, and *S. vermiculata* [26], suggesting that selected *Salsola* extracts may offer mild postprandial glycemic control. These inhibitory patterns indicate that the difference is not only by solvent but also tightly linked to species-specific phytochemical composition. *S. ruthenica*, *S. kali*, and *S. stenoptera* emerge as the most promising candidates for enzyme-targeted applications, particularly in neurodegenerative and dermatological contexts. The elevated activities in EA and MeOH extracts are consistent with the presence of mid-polar phenolic compounds and amide alkaloids, as also indicated by VIP markers in the metabolomic dataset (see Section 3.4).

### 2.4. Untargeted Metabolomics and Multivariate Modeling: Identifying Chemotaxonomic Markers

To comprehensively evaluate the phytochemical diversity across five *Salsola* species and three different extraction solvents, an untargeted metabolomic approach was conducted using UHPLC-ESI-QTOF-MS, and enabled the annotation of 265 metabolites, as detailed in the Supplementary Material (Table S1). The identified compounds encompassed a wide spectrum of phytochemical classes, including flavonoids (anthocyanins, flavanols, and flavones), phenolic acids, lignans, stilbenes, and other low-molecular-weight bioactives, reflecting the chemical complexity inherent to the *Salsola* genus.

To unravel patterns of similarity and dissimilarity in the metabolite composition across the samples, the full dataset was subjected to multivariate statistical analyses. This included both unsupervised (Hierarchical Cluster Analysis, HCA) and supervised methods (Analysis of Variance Multiblock OPLS, AMOPLS).

**Figure 1:**
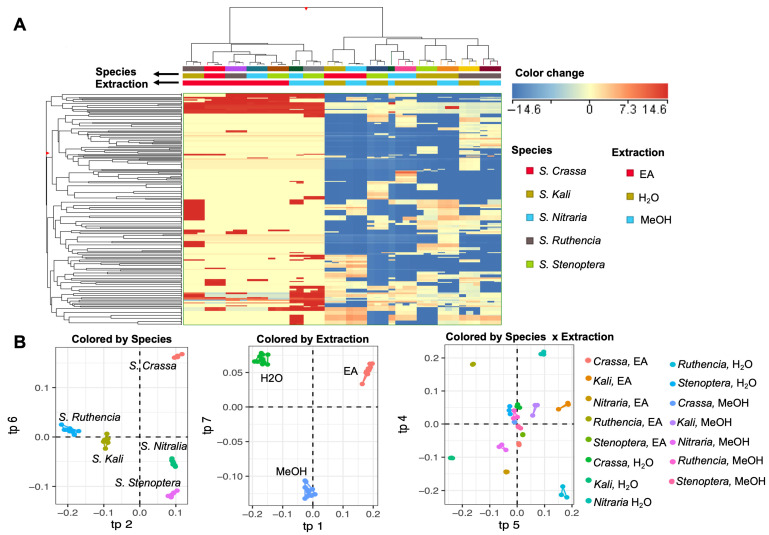
(**A**) Hierarchical clustering analysis (HCA) of the untargeted metabolomic profile of five *Salsola* species extracted using ethyl acetate (EA), water (H_2_O), and methanol (MeOH). Clustering was performed based on the fold change heatmap, using median values of all samples as the baseline (Euclidean distance, Ward’s algorithm). (**B**) AMOPLS score plot illustrating the influence of statistically significant factors: Species, Extraction, and their interaction (Species × Extraction).

The HCA revealed that sample clustering was not strictly dictated by species alone, as clusters contained mixed species profiles (Figure 1A). This lack of distinct species-based clustering suggests a level of phytochemical overlap among certain species, potentially due to shared ecological adaptations or phylogenetic proximity. Nonetheless, some degree of species-level distinction was evident, indicating that species identity still plays a substantial role in determining phytochemical content [27]. In line with expectations from plant secondary metabolism, natural variation driven by genetics and environmental factors leads some species to accumulate higher levels or unique profiles of specific compounds. In contrast, the extraction solvent appeared to exert a more defined influence on sample clustering. Notably, EA-derived extracts often formed a separate cluster from those obtained using MeOH or H_2_O. This separation aligns with the solvents’ differing polarities, EA being relatively non-polar and more selective for mid- to non-polar compounds such as certain alkaloids, terpenoids, and some lignans, while MeOH and H_2_O are more efficient at extracting polar and hydrophilic compounds like phenolics and flavonoids [28]. These results emphasize that solvent polarity is a major determinant of extraction efficiency and compound selectivity. Further insight was obtained from the heat map, which visualized the distribution of compound classes across the species–solvent combinations. Here, specific combinations, such as *S. stenoptera* EA, stood out with distinct phytochemical signatures, demonstrating that the extraction effect is not uniform across species. Instead, a significant species × extraction interaction was evident, highlighting the complex interplay between plant matrix and solvent characteristics. In practical terms, this suggests that the optimal extraction solvent for a given phytochemical class may vary depending on the plant species, reinforcing the importance of method optimization in metabolomic studies [18,29].

A supervised AMOPLS model was performed to quantitatively evaluate the contributions of each experimental factor to the observed variation (Figure 1B and Figure S1). This multivariate analysis allows for the decomposition of the total variability into components attributable to species, solvent, and their interaction. The AMOPLS results demonstrated that all three factors significantly impacted the metabolite composition. Specifically, species and the species × extraction interaction accounted for 35% and 36% of the total variance, respectively, while extraction alone explained 21% (Table S2). These findings underscore the dominant role of species-specific metabolic traits and their dynamic response to extraction conditions in shaping the chemical landscape. To further dissect the multivariate patterns observed across the *Salsola* metabolomes, Variable Importance in Projection (VIP^2^) scores were extracted from the AMOPLS model (Table 4 and Table S3). These VIP markers reflect the most influential compounds contributing to the main effects of species and extraction, as well as their interaction, thereby linking observed statistical separation to specific metabolic features. The VIP markers associated with the species effect revealed a strong contribution from flavonoids, lignans, phenolic acids, and anthocyanins, highlighting distinct biosynthetic capacities across the five *Salsola* species.

Among the most species-discriminant metabolites were: p-Coumaroyl glycolic acid and rosmarinic acid, both well-known phenolic derivatives, suggesting differential activity in the phenylpropanoid pathway across species [30]. Cirsimaritin, hispidulin, chrysoeriol derivatives, and spinacetin 3-O-glucosyl-(1→6)-[apiosyl(1→2)]-glucoside, flavonoids that are often linked to stress adaptation and antioxidant defense, were particularly abundant in certain species. Secoisolariciresinol, matairesinol, and pinoresinol were key lignans contributing to species-level variation, indicating species-specific allocation toward lignan biosynthesis. A variety of glycosylated quercetin and kaempferol derivatives (e.g., kaempferol 3-O-glucosyl-rhamnosyl-galactoside, quercetin 3-O-glucoside, and isorhamnetin 3-O-glucoside 7-O-rhamnoside) also played central roles, likely reflecting genotypic differences in flavonol glycosylation patterns. The presence of anthocyanin conjugates such as pelargonidin 3-O-glucosyl-rutinoside and delphinidin 3-O-feruloyl-glucoside further supports pigment-related specialization among species, possibly related to flower/leaf color or UV protection.

Regarding the extraction solvent effect, the VIP markers reflected the chemical polarity of the extracted compounds, aligning well with the solvent properties. Phenolic acids such as gallic acid, cinnamic acid, ferulic acid 4-O-glucoside, and m-coumaric acid showed higher extraction in polar solvents like MeOH and H_2_O, supporting their solubility in hydrophilic matrices [31]. Dihydrosterculic acid, lactobacillic acid, and nonadecanoic acid, medium-chain fatty acids and fatty acid derivatives, were preferentially extracted by EA, a less polar solvent, underlining its affinity for lipophilic constituents [32]. Flavonoids with diglycosidic or triglycosidic substitutions (e.g., kaempferol 3,7-O-diglucoside, quercetin 3-O-rhamnosyl-galactoside) were also selectively recovered based on solvent, highlighting how polarity influences the recovery of complex glycosides. Volatile or semi-volatile phenolics such as carvacrol, thymol, 4-vinylguaiacol, and 4-vinylphenol were among the key discriminants favoring EA extraction, which is consistent with their moderate lipophilicity and volatility. These patterns reinforce the principle that solvent polarity directly shapes the metabolomic fingerprint, and solvent choice must be aligned with compound class to ensure optimal extraction, an especially important consideration in quality control and phytochemical standardization [19].

The species × extraction interaction effect, which accounted for the largest portion of variance (36%), identified VIP markers whose abundance was not explained by species or solvent alone, but rather by their combination. This means certain compounds were highly expressed only under specific extraction conditions in specific species. 5,6-Dihydroxy-7,8,3′,4′-tetramethoxyflavone, 3-methoxynobiletin, and eriodictyol, flavonoids linked to stress and defense pathways, emerged as high VIP markers only in specific species-solvent combinations, further emphasizing this conditional expression. Steroidal ferulates (e.g., sitosterol ferulate, schottenol ferulate) and resorcinol derivatives (5-tricosylresorcinol, 5-heneicosylresorcinol) were also highly interaction-specific, reflecting compound classes that are sensitive to both species’ metabolic background and solvent affinity. Delphinidin and cyanidin derivatives, including delphinidin 3-O-sambubioside and cyanidin 3-O-glucosyl-rutinoside, further highlighted pigment biosynthesis as a highly condition-dependent trait, linked to species physiology and extraction efficiency.

The identification of VIP markers through AMOPLS offers deep insights into the metabolomic markers of species differentiation, solvent selectivity, and their interaction. While species-level variation was most pronounced in flavonoid and lignan content, solvent polarity governed the preferential extraction of phenolic acids, volatiles, and fatty acid derivatives. Most compellingly, the species × solvent interaction revealed that certain metabolites are only detectable under specific extraction conditions for certain species, emphasizing the need for targeted extraction strategies in phytochemical analysis and natural product discovery. These discriminative metabolites not only support the multivariate statistical patterns but also provide chemotaxonomic markers and potential bioactivity leads, contributing to a more nuanced understanding of *Salsola* phytochemistry.

### 2.5. Semi-Quantitative Profiling of Phenolic Classes: Composition Trends and Extractability

The semi-quantification of the phenolic profile obtained from the annotated chemical entities revealed a diverse phenolic composition distributed in several major classes and sub-classes, and showed that the flavonoid class was the most prominent, with 82 compounds identified, including anthocyanins, flavanones, flavonols, and flavanols. The remaining compounds belonged to phenolic acids, lignans, low-molecular-weight (LMW) phenolics, and other classes (Table S3).

A two-way multivariate analysis of variance (MANOVA) was conducted to assess the effects of *Salsola* species, extraction solvent, and their interaction on the quantified classes of phenolic compounds. The multivariate test revealed that all main effects (species and extraction) and their interaction were highly significant (*p* < 0.001), indicating that both factors, independently and in combination, significantly influence the overall phenolic composition (Table S4). Univariate ANOVA results further showed that almost all phenolic classes were significantly affected (*p* < 0.001) by species, extraction solvent, and their interaction (Table 5). Notably, anthocyanins, flavanols, flavones, flavonols, lignans, low-molecular-weight phenolics (LMW), and phenolic acids showed strong variation among species and solvents. The significant interaction effects suggest that the extraction efficiency of each solvent varied depending on the species, emphasizing the importance of matching solvent type to plant species for optimal recovery of specific phenolic classes. The results highlight that phenolic composition in *Salsola* species is highly species-specific and extraction-dependent. The clear interaction between species and solvent underscores the need for tailored extraction strategies when profiling bioactive phenolics across diverse plant species.

The comparative analysis of phenolic profiles across five *Salsola* species revealed distinct metabolic specializations, significantly influenced by both species and solvent type. When aggregating the data across solvents (Table S4), *S. ruthenica* emerged as the species with the richest phenolic composition overall (Table S5). It had the highest concentrations of low-molecular-weight (LMW) phenolics (4420.7 mg/kg DW), flavanols (141.6 mg/kg DW), and phenolic acids (288.8 mg/kg DW). Notably, its LMW content was over 10-fold higher than that of *S. crassa* (463.9 mg/kg DW), highlighting its strong antioxidant capacity. *S. nitraria* also showed remarkable LMW accumulation, with an average of 4148.5 mg/kg DW. This exceptionally high content was primarily attributed to water extraction, where values peaked at 10,508.1 mg/kg DW, suggesting water as an ideal solvent for hydrophilic phenolics in this species. The dramatic enhancement in LMW phenolics by water extraction in *S. nitraria* illustrates how species-specific metabolic traits can synergize with solvent polarity to yield targeted phytochemical enrichment. This is consistent with findings in other plant systems. For example, a study reported that solvent polarity significantly influences total phenolic content: water extracts (Polarity Index = 9.0) outperformed less polar solvents for *Isatis tinctoria*, highlighting water’s ability to enrich polar phenolic compounds selectively [19]. In contrast, *S. stenoptera* and *S. kali* presented more moderate LMW values, yet *S. stenoptera* demonstrated outstanding flavanol levels (143.9 mg/kg DW), tying it with *S. ruthenica* as the most flavanol-rich species. In terms of flavones, *S. kali* was the most enriched species, averaging 118.2 mg/kg DW, which was nearly six times higher than that observed in *S. nitraria*. Similarly, *S. crassa* and *S. ruthenica* followed with moderate levels, whereas *S. stenoptera* had intermediate accumulation. *S. kali* also demonstrated strong flavonol content (289.8 mg/kg DW), which aligns with its high flavone levels and supports a more diverse flavonoid profile. These chemotaxonomic differences suggest that certain *Salsola* species may have evolved preferential biosynthetic routes within the flavonoid pathway, offering potential as sources of specialized nutraceutical ingredients.

Looking at the influence of extraction solvents (Table 2), methanol (MeOH) proved to be the most effective across nearly all phenolic classes. It yielded the highest values for anthocyanins, flavanols, flavones, flavonols, and phenolic acids. Compared to water, methanol extraction increased flavonol yield by over 6-fold (348.2 vs. 55.2 mg/kg DW) and flavone yield by nearly 5-fold. This underscores MeOH’s broad-spectrum extraction capacity for both polar and moderately non-polar phenolics. These results are consistent with previous studies that identify methanol as one of the most universally effective solvents for phenolic recovery, particularly when targeting a broad polarity range [17,20]. EA, in contrast, showed high specificity for more lipophilic compounds. It extracted the greatest amount of lignans (767.7 mg/kg DW), approximately 8-fold higher than that achieved with water (94.4 mg/kg DW), and over 4-fold higher than with methanol. It also favored phenolic acid extraction, indicating its selectivity for less polar phenolic classes.

On the species-solvent interaction level (Table 5), some combinations stood out distinctly. The *S. ruthenica*–methanol pair yielded extremely high levels of flavones (226.4 mg/kg DW), flavonols (329.2 mg/kg DW), and lignans (821.9 mg/kg DW), suggesting the synergistic effect of this species-solvent combination. In *S. nitraria*, the water extract was dominated by LMW phenolics, reinforcing the hypothesis that this species is particularly adept at accumulating water-soluble metabolites. *S. crassa*, although not the richest in overall phenolics, had a notably high content of phenolic acids and lignans, particularly in EA extracts. Its MeOH extract also revealed the highest anthocyanin concentration (153.5 mg/kg DW), which was not detectable in EA and significantly higher than its water-based counterpart (17.8 mg/kg DW). In addition, *S. stenoptera* showed a relatively narrower phenolic profile, with a clear dominance in flavanols (233.7 mg/kg DW in MeOH) and LMW phenolics (1438.9 mg/kg DW in MeOH). Despite its modest anthocyanin and phenolic acid contents, the elevated levels of these two phenolic groups suggest potential niche applications in health-promoting formulations.

### 2.6. Bioactivity-Phytochemical Correlations: Functional Links Between Compounds and Effects

**Figure 2:**
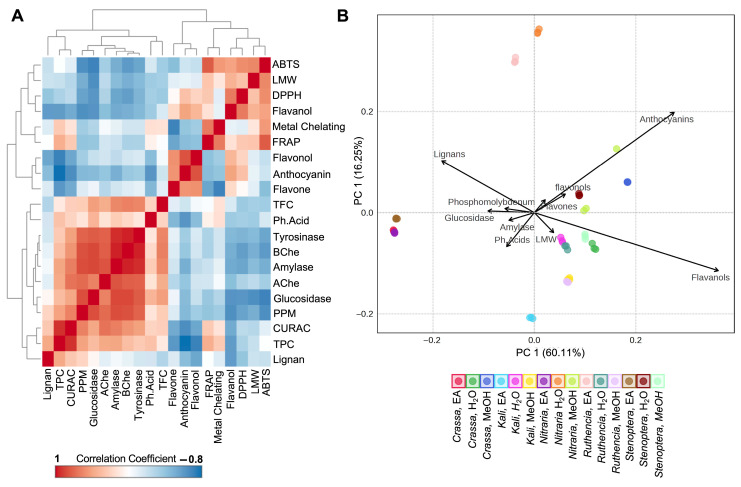
(**A**) Pearson correlation matrix highlighting the relationship between total bioactive components, biological activities, and phenolic subclasses. (**B**). Principal component analysis (PCA) biplot showing the distribution of total bioactive compounds, biological activities, and phenolic subclasses. Arrows indicate the contribution and direction of each variable to the principal components. Abbreviations: ABTS, 2,2′-azino-bis(3-ethylbenzothiazoline) 6-sulfonic acid; LMW, low molecular weight compounds; CUPRAC, cupric ion reducing antioxidant capacity; DPPH, 2,2-diphenyl-1-picrylhydrazyl; FRAP, ferric ion reducing antioxidant power; Ph. Acid, phenolic acids; BChe, butyrylcholinesterase; AChe, acetylcholinesterase; PPM, phosphomolybdenum activity; TPC, total phenolic acid content; TFC, total flavonoid content.

Accordingly, Pearson’s correlation analysis was performed to explore the relationships among all annotated classes of bioactive compounds, as well as antioxidant and enzyme inhibitory activities (Figure 2A, Table S6). The correlation matrix reveals distinct patterns among phytochemical classes and their associated bioactivities across the *Salsola* species studied. Antioxidant assays such as FRAP, ABTS, and DPPH are highly interrelated, particularly with strong positive correlations observed between FRAP and ABTS (r = 0.81), and between DPPH and flavanols (r = 0.70). These results suggest that flavanols significantly contribute to the antioxidant capacity, especially as measured by electron transfer-based assays. This suggests that flavanols, due to their hydroxylation pattern and redox potential, are especially effective at scavenging radicals through electron transfer mechanisms. Their correlation with multiple antioxidant assays reinforces their role as core antioxidant agents in the phenolic pool [16,33]. Total phenolic content (TPC) emerges as a major determinant of antioxidant activity, showing a particularly strong correlation with CUPRAC (r = 0.93) and moderate correlations with FRAP and enzyme inhibition activities such as AChE and amylase. Interestingly, anthocyanins and flavonols, which are highly correlated with each other (r = 0.84), display strong negative correlations with TPC (r = −0.82 and −0.72, respectively) and several antioxidant assays, indicating that species rich in these pigments may follow distinct biosynthetic or metabolic profiles compared to those high in general phenolics. This antagonistic trend between pigmented flavonoids and total phenolics may reflect a metabolic trade-off or divergence in resource allocation, where certain species prioritize pigmentation over antioxidant function, possibly linked to ecological or developmental adaptations [34]. Enzyme inhibition assays also cluster tightly together, with very high correlations observed among AChE, BChE, tyrosinase, and amylase (r > 0.90), suggesting common bioactive compounds may drive multi-target inhibition. Lignans, while moderately correlated with TPC (r = 0.53), exhibit negative correlations with anthocyanins, flavonols, and flavanols, indicating their abundance in phytochemical profiles that are distinct from those rich in flavonoid subclasses. The findings support the existence of distinct chemotypes among the studied *Salsola* species, where certain phytochemical classes preferentially contribute to specific antioxidant or enzyme inhibitory effects.

Principal Component Analysis (PCA) was employed to uncover the primary patterns of variation and interrelationships among the semi-quantified phenolic subclasses and bioactivity parameters across the samples. The first two principal components (PC1 and PC2) accounted for a cumulative 76.37% of the total variance. The biplot revealed that anthocyanins and flavanols exhibited the longest vectors, indicating their dominant contribution to the total variance and their strong influence on sample distribution along the principal components (Figure 2B). Their prominence is consistent with their highly variable distribution across species and solvents. For example, *S. crassa*-MeOH and *S. kali*-MeOH showed exceptionally high anthocyanin levels, while *S. ruthenica* and *S. stenoptera* displayed the highest flavanol concentrations (Table 5). Lignans and phenolic acids also showed notable contributions, playing a secondary yet meaningful role in differentiating the samples. Several enzyme inhibition assays, particularly glucosidase and amylase, along with phosphomolybdenum antioxidant capacity, aligned closely with certain phytochemical groups, implying functional associations between specific phenolic classes and biological activities. In contrast, flavonols, low-molecular-weight phenolics (LMW), and flavones contributed less to the observed variance. The PCA highlights a strong interplay between distinct phenolic profiles and biofunctional potentials, offering insight into the compounds that may serve as key chemotaxonomic or bioactivity markers.

**Figure 3:**
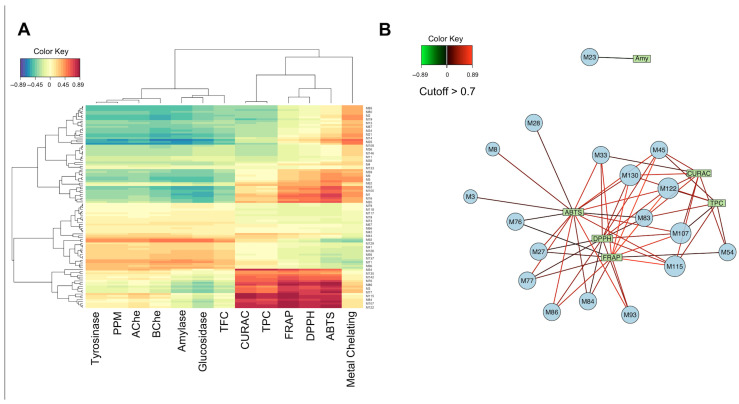
Correlation heatmap (**A**) illustrating the relationships between metabolites and bioassays, and relevance network plot (**B**) highlighting the most strongly correlated features (r > 0.7) between metabolites and biological activities. Metabolite names corresponding to these compounds are provided in Supplementary Table S3.

Finally, regularized canonical correlation analysis (rCCA) was employed to explore statistical associations between metabolites and bioactivity. The rCCA scores were visualized in a heatmap, with high-correlation latent variables further mapped in a network diagram (Figure 3). ABTS and DPPH activities showed positive associations with several glycosylated flavonoids, including Quercetin 3-O-glucosyl-xyloside, Sakuranetin, and p-Coumaroyl glycolic acid. These compounds are known for their strong hydrogen-donating ability and ability to stabilize free radicals through extended conjugation and glycosylation, which enhances solubility and cellular uptake [35]. Sakuranetin, a methylated flavanone, has demonstrated antioxidant activity through radical scavenging and anti-inflammatory effects via MAPK and STAT1 pathway modulation [36]. These properties support its observed association with ABTS and DPPH activity in our study. CUPRAC and FRAP, which measure reducing power rather than radical scavenging, were associated with quercetin 3-O-glucosyl-xyloside and 6″-O-acetylgenistin, an acetylated isoflavone. These compounds possess multiple hydroxyl groups and extended aromatic systems that facilitate electron donation to metal ions, a core mechanism suggesting their reducing capacity. Additionally, ABTS activity correlated positively with lignans, including secoisolariciresinol, anhydro-secoisolariciresinol, medioresinol, and *p*-coumaric acid ethyl ester. These lignans are known to contribute significantly to antioxidant activity via both radical scavenging and metal ion chelation, particularly in plant species adapted to oxidative stress environments [37]. Their prominent role in our correlation models supports their potential function as key redox mediators in *Salsola* extracts. The rCCA findings suggest that glycosylated flavonoids and lignans are associated with antioxidant potential in *Salsola* species. The structural features of these compounds, such as hydroxylation, glycosidic substitution, and aromatic conjugation, are closely linked to their capacity for hydrogen atom transfer and single-electron transfer mechanisms, which suggest the modes of action in ABTS, DPPH, FRAP, and CUPRAC assays [38].

## 3. Materials and Methods

### 3.1. Plant Material and Extraction Procedure

In 2019, five *Salsola* species (*S. crassa*, *S. kali*, *S. nitraria*, *S. ruthenica* and *S. stenoptera*) were collected from different regions of Turkey, with location details available in the supplementary materials (Table S7). Dr. Evren Yildiztugay executed the taxonomic identification of these specimens. A voucher specimen was officially lodged in the Selcuk University herbarium. Post-collection, the aerial parts of the plant were meticulously separated and shade-dried at room temperature to maintain their phytochemical properties.

The powdered plant material was used for extraction for both functional bioassays and untargeted metabolomics, following protocol-specific procedures. For bioassay analyses, three distinct solvents, ethyl acetate, methanol, and water, were used to facilitate the extraction of bioactive compounds from the plant material. For organic solvent-based extraction, a 10-gram sample was subjected to maceration in 200 mL of ethyl acetate and methanol for 24 h at ambient temperature (25 ± 2 °C). In contrast, aqueous extraction was performed by infusing 10 g of the plant material in hot distilled water (80–90 °C) for 15 min under gentle agitation. Post-extraction, the aqueous extract was concentrated through freeze-drying (lyophilization) to obtain a dry powder, while residual organic solvents were removed from the ethyl acetate and methanol extracts using rotary evaporation at reduced pressure (40–60 mbar, 40–50 °C). The obtained extracts were then used for untargeted metabolomics with further resuspension in the mentioned solvents.

### 3.2. Total Phenolic and Flavonoid Contents

The Folin–Ciocalteu and AlCl_3_ assays, respectively, were utilized to determine the total phenolic and flavonoid contents, and the procedures have been previously reported [39].

### 3.3. Non-Enzymatic Antioxidant Activities

To assess the antioxidant potential in leaf extracts, a set of six complementary in vitro spectrophotometric tests was performed. These included the ABTS and DPPH (which examine the ability to neutralize free radicals), FRAP and CUPRAC (which evaluate the reduction capabilities), as well as metal chelating ability (MCA) and phosphomolybdenum (PPM) assays. Each assay was evaluated using Trolox as a reference standard, except for MCA, which was expressed in terms of EDTA equivalent per gram of extract. All the procedures used are given in our previous work [39].

### 3.4. Inhibitory Effects Against Some Key Enzymes

Enzyme inhibition effects were evaluated following the spectrophotometric protocol established by Grochowski et al. [40]. Cholinesterase inhibition potentials (AChE and BChE) were standardized against galanthamine hydrobromide and expressed as milligram galanthamine equivalents per gram dry extract (mg GALAE/g). Carbohydrate-hydrolyzing enzyme inhibition (α-amylase and α-glucosidase) was quantified relative to acarbose standard (mmol ACAE/g). Tyrosinase inhibition capacity is reported as milligram kojic acid equivalents per gram (mg KAE/g).

### 3.5. Bioactive Compounds Profiling Using Untargeted Metabolomics

The untargeted metabolomics analysis was performed as described [41]. To evaluate solvent-dependent variations in phytochemical profiles, the same extracts as described in Section 3.1 were resuspended (1:10, *w*/*v*) in (1) distilled water, (2) ethyl acetate, and (3) 0.1% formic acid in 80% aqueous methanol, and then centrifuged at 7197× *g* for 15 min at 4 °C. The supernatants were filtered through 0.22 μm syringe filters to remove any remaining particulates. The analysis was carried out using ultra-high-pressure liquid chromatography coupled with a quadrupole-time-of-flight mass spectrometer (UHPLC-ESI-QTOF-MS), featuring a Dual JetStream Electrospray Ionization System (all from Agilent Technologies, Santa Clara, CA, USA). For each sample, 6 μL was injected into a 1290 liquid chromatograph (column: 2.1 × 100 mm, 1.9 μm) connected to a G6550 mass spectrometer detector. Chromatography was performed using a water-acetonitrile reverse phase gradient elution (6–94% acetonitrile over 33 min), and mass spectrometry employed positive polarity SCAN acquisition, covering a range of 100–1200 *m*/*z*.

For the analysis of bioactive compounds, raw mass feature processing and compound annotation were performed using the Agilent Profinder B.10.0 software (Agilent Technologies) against the Phenol-Explorer database [42]. Metabolite annotation was carried out by integrating monoisotopic accurate mass with isotopic pattern, including isotope spacing and relative abundance, using a mass accuracy tolerance of <5 ppm. Annotation confidence adhered to Level 2 of the COSMOS metabolomics standards, corresponding to putatively identified compounds based on physicochemical properties and spectral similarity with public databases [43]. Only compounds detected in at least two of three technical replicates per sample were considered for further analysis. In parallel, a pooled quality control (QC) sample was prepared by combining equal aliquots from all individual samples. The QC sample was injected periodically throughout the analytical sequence, specifically at the beginning, midpoint, and end of the run, to monitor system stability and reproducibility. The QC sample was further subjected to data-dependent MS/MS fragmentation (N = 9) using collision energies 10, 20, and 40 eV.

Semi-quantitative analysis of the annotated compounds was performed by categorizing them into their respective chemical classes. Each subclass was quantified relative to a representative analytical standard from the same class, which was analyzed under identical experimental conditions. This approach followed a previously established methodology, enabling consistent cross-comparison of compound abundance across different treatments [44]. Calibration curves for all reference standards (purchased from Merck KGaA, Darmstadt, Germany) were constructed within a concentration range of 6.25 to 500 mg/L. The following standards were used to represent specific chemical subclasses of metabolites, each analyzed under identical conditions: sesamin for lignans (y = 229,159x, R^2^ = 0.993); ferulic acid for phenolic acids (y = 457,774x, R^2^ = 0.999); cyanidin for anthocyanins (y = 599,208x, R^2^ = 0.998); tyrosol for low molecular weight compounds (abbreviated as LMW, including coumarins, tyrosols, phenolic aldehydes and alkylphenols) (y = 258,779x, R^2^ = 0.986); (+)-catechin for flavanols (y = 790,261x, R^2^ = 0.997); quercetin for flavonols (y = 1,090,960x, R^2^ = 0.987); and luteolin for flavones and other flavonoids, including flavanones and isoflavonoids (y = 3,000,000x, R^2^ = 0.988); ferulic acid for phenolic acids (y = 457,774x, R^2^ = 0.991). The content of each subclass was expressed as equivalents of the corresponding reference standard in µg g^−1^ of dry extract.

### 3.6. Statistical and Multivariate Analysis

The statistical analysis for metabolomics was performed using the Mass Profiler Professional 15.1 software (Agilent Technologies, Santa Clara, CA, USA). The samples were previously transformed using a log2 transformation and then normalized and baselined against the median abundance. Afterward, two approaches—unsupervised (hierarchical cluster analysis using Euclidean distance, based on fold-change heatmaps) and supervised (Orthogonal projections to latent structures-discriminant analysis, OPLS-DA)—were combined to analyze and interpret the data. ANOVA (*p* ≤ 0.05, Benjamini–Hochberg correction) and fold-change (FC > 2.0) analysis were combined in Volcano Plot analysis to identify differential compounds among the different treatments. To further investigate the effect of experimental factors (species type and extraction method) and their interactions on the metabolomics data, a supervised analysis of variance multiblock orthogonal partial least squares (AMOPLS) approach was employed. This analysis was performed in R software (version 4.2.3) using the rAMOPLS package [45]. Model validation and statistical significance were assessed through 100 random permutation tests. The results were expressed using key metrics: relative sum of squares (RSS), residual structure ratio (RSR), *p*-values, and principal predictive components. Variable Importance in Projection (VIP^2^) scores were calculated to pinpoint metabolites most responsible for group discrimination, and variables with the highest VIP values were identified as key contributors to the observed effects.

The semi-quantitative data were statistically analyzed to assess the effects of different *Salsola* species and extraction methods. Both one-way and two-way analyses of variance (ANOVA) were conducted at a significance level of *p* < 0.05. Where significant differences were observed, Duncan’s post hoc test was applied to determine specific pairwise comparisons among groups. Pearson’s correlation analysis and PCA biplot were carried out using MetaboAnalyst 6.0 (https://www.metaboanalyst.ca/ (accessed on 10 November 2025)) to examine the correlation between different phenolic classes and biological activities. Regularized Canonical Correlation Analysis (rCCA) was performed using the *mixOmics* package in R.

## 4. Conclusions

This study provides a multidimensional understanding of how intrinsic (species-specific) and extrinsic (extraction-related) factors synergistically influence the phytochemical composition of *Salsola* species. Rather than treating species or extraction as isolated variables, our findings reveal their profound interdependence in shaping both the chemical fingerprints and the bioactive potential of plant extracts. Methanol emerged as the most efficient solvent across phenolic subclasses, while ethyl acetate and water showed distinct selectivity for specific compounds, highlighting the value of tailored extraction strategies. Additionally, *S. ruthenica* and *S. nitraria* were found as the most promising species for phenolic exploitation, each with distinct solvent preferences and compound profiles. The integration of chemical profiling with bioassay activities, supported by multivariate statistical analyses, allowed for the identification of species- and solvent-associated chemotaxonomic markers and suggested exploratory associations between specific metabolite classes, such as flavanols, lignans, and glycosylated quercetin derivatives, and bioactivities. These insights pave the way for the targeted development of natural antioxidants and enzyme inhibitors, positioning *Salsola* as a versatile and underexplored genus in the quest for plant-based antioxidants, enzyme inhibitors, and nutraceutical ingredients.

## Figures and Tables

**Table 1:** Total phenolic (TPC) and flavonoid (TFC) contents in aerial parts extracts of *Salsola* species using ethyl acetate, methanol, and water as solvents.

Species	Extracts	TPC (mg GAE/g)	TFC (mg RE/g)
*S. crassa*	EA	21.78 ± 0.60 ^e^	14.38 ± 0.14 ^c^
	MeOH	14.55 ± 0.32 ^g^	14.12 ± 0.22 ^c^
	H_2_O	11.05 ± 0.22 ^i^	3.01 ± 0.14 ^j^
*S. kali*	EA	25.19 ± 0.54 ^d^	17.33 ± 0.14 ^b^
	MeOH	22.64 ± 0.69 ^e^	20.50 ± 0.01 ^a^
	H_2_O	13.57 ± 0.23 ^gh^	4.15 ± 0.08 ^i^
*S. nitraria*	EA	23.84 ± 0.43 ^de^	2.85 ± 0.32 ^j^
	MeOH	11.89 ± 0.57 ^hi^	13.21 ± 0.16 ^d^
	H_2_O	12.11 ± 0.18 ^hi^	4.43 ± 0.12 ^hi^
*S. ruthenica*	EA	27.94 ± 0.22 ^b^	10.55 ± 0.17 ^e^
	MeOH	46.04 ± 2.24 ^a^	10.47 ± 0.21 ^ef^
	H_2_O	25.68 ± 0.26 ^cd^	2.57 ± 0.02 ^j^
*S. stenoptera*	EA	27.32 ± 0.20 ^bc^	9.83 ± 0.46 ^fg^
	MeOH	19.07 ± 0.35 ^f^	9.76 ± 0.45 ^g^
	H_2_O	11.34 ± 0.11 ^i^	5.10 ± 0.03 ^h^

Values are reported as mean ± SD of three technical replications. GAE: Gallic acid equivalents; RE: Rutin equivalents. Different letters in the same column indicate significant differences in the extracts (*p* < 0.05).

**Table 2:** Antioxidant capacities of aerial parts extracts of *Salsola* species obtained using ethyl acetate, methanol, and water.

Species	Extracts	DPPH (mg TE/g)	ABTS (mg TE/g)	CUPRAC (mg TE/g)	FRAP (mg TE/g)	MCA (mg EDTAE/g)	PPM (mmol TE/g)
*S. crassa*	EA	2.88 ± 0.76 ^k^	18.12 ± 1.58 ^k^	51.16 ± 1.34 ^f^	24.47 ± 0.27 ^fg^	19.79 ± 0.84 ^bc^	1.20 ± 0.06 ^b^
	MeOH	19.01 ± 0.66 ^e^	34.96 ± 0.85 ^g^	52.54 ± 0.77 ^ef^	30.05 ± 0.61 ^e^	18.47 ± 0.51 ^cd^	0.46 ± 0.02 ^ef^
	H_2_O	12.06 ± 0.04 ^g^	42.68 ± 0.58 ^ef^	31.18 ± 0.11 ^hi^	26.49 ± 0.54 ^f^	21.19 ± 0.35 ^ab^	0.07 ± 0.01 ^g^
*S. kali*	EA	8.06 ± 0.40 ^hi^	26.90 ± 0.33 ^i^	71.92 ± 2.39 ^b^	34.92 ± 1.01 ^d^	17.08 ± 0.69 ^de^	1.59 ± 0.07 ^a^
	MeOH	30.19 ± 0.86 ^c^	52.06 ± 1.35 ^c^	76.08 ± 0.69 ^b^	46.40 ± 1.20 ^b^	17.93 ± 0.60 ^cde^	0.62 ± 0.03 ^d^
	H_2_O	12.56 ± 0.27 ^g^	40.50 ± 2.25 ^f^	37.48 ± 0.72 ^gh^	26.56 ± 0.40 ^f^	21.05 ± 0.07 ^ab^	0.10 ± 0.01 ^g^
*S. nitraria*	EA	4.92 ± 0.48 ^j^	30.09 ± 0.72 ^h^	59.57 ± 3.20 ^de^	24.80 ± 1.00 ^fg^	16.48 ± 0.52 ^e^	1.54 ± 0.04 ^a^
	MeOH	6.85 ± 0.33 ^i^	33.11 ± 0.59 ^gh^	41.12 ± 0.91 ^g^	22.69 ± 0.39 ^g^	20.88 ± 1.78 ^ab^	0.44 ± 0.03 ^f^
	H_2_O	8.37 ± 0.21 ^hi^	53.14 ± 0.71 ^c^	27.86 ± 0.23 ^i^	30.98 ± 0.60 ^e^	21.96 ± 0.04 ^a^	0.06 ± 0.01 ^g^
*S. ruthenica*	EA	14.80 ± 0.41 ^f^	46.23 ± 0.05 ^d^	72.76 ± 3.65 ^b^	38.82 ± 0.87 ^c^	13.81 ± 0.45 ^f^	1.58 ± 0.01 ^a^
	MeOH	47.21 ± 0.09 ^a^	97.40 ± 0.77 ^a^	141.38 ± 6.29 ^a^	80.30 ± 1.12 ^a^	19.78 ± 0.26 ^bc^	0.95 ± 0.11 ^c^
	H_2_O	35.18 ± 0.43 ^b^	83.74 ± 0.74 ^b^	69.49 ± 1.09 ^bc^	47.71 ± 0.32 ^b^	20.89 ± 0.05 ^ab^	0.46 ± 0.03 ^ef^
*S. stenoptera*	EA	7.59 ± 0.99 ^hi^	22.95 ± 1.56 ^j^	63.37 ± 2.89 ^cd^	29.20 ± 0.96 ^e^	18.50 ± 0.65 ^cd^	1.17 ± 0.07 ^b^
	MeOH	21.48 ± 0.51 ^d^	51.84 ± 0.59 ^c^	57.54 ± 1.07 ^def^	38.40 ± 0.97 ^c^	21.02 ± 0.24 ^ab^	0.59 ± 0.05 ^de^
	H_2_O	8.64 ± 0.37 ^h^	45.41 ± 0.75 ^de^	29.29 ± 0.16 ^i^	35.01 ± 1.53 ^d^	21.32 ± 0.05 ^ab^	0.06 ± 0.01 ^g^

Values are reported as mean ± SD of three technical replications. PPM: Phosphomolybdenum; MCA: Metal chelating Activity; TE: Trolox Equivalent; EDTAE: EDTA equivalent. Different letters in the same column indicate significant differences in the extracts (*p* < 0.05).

**Table 3:** Enzyme inhibitory effects of *Salsola* aerial part extracts against AChE, BChE, tyrosinase, α-amylase, and α-glucosidase.

Species	Extracts	AChE (mg GALAE/g)	BChE (mg GALAE/g)	Tyrosinase (mg KAE/g)	Amylase (mmol ACAE/g)	Glucosidase (mmol ACAE/g)
*S. crassa*	EA	1.45 ± 0.06 ^e^	2.32 ± 0.07 ^ab^	56.33 ± 0.48 ^a^	0.45 ± 0.02 ^c^	0.95 ± 0.04 ^ab^
	MeOH	1.95 ± 0.13 ^cd^	1.89 ± 0.02 ^cd^	54.42 ± 2.89 ^a^	0.32 ± 0.01 ^de^	0.35 ± 0.01 ^e^
	H_2_O	0.56 ± 0.04 ^g^	na	na	0.04 ± 0.01 ^g^	na
*S. kali*	EA	2.11 ± 0.05 ^bcd^	2.21 ± 0.10 ^abc^	27.33 ± 2.24 ^c^	0.45 ± 0.01 ^c^	0.89 ± 0.01 ^c^
	MeOH	2.25 ± 0.07 ^ab^	1.57 ± 0.10 ^d^	48.56 ± 1.94 ^ab^	0.26 ± 0.01 ^f^	1.00 ± 0.01 ^a^
	H_2_O	0.66 ± 0.09 ^g^	na	na	0.04 ± 0.01 ^g^	na
*S. nitraria*	EA	2.17 ± 0.17 ^abc^	2.00 ± 0.01 ^bc^	45.27 ± 3.16 ^b^	0.50 ± 0.03 ^b^	0.86 ± 0.06 ^c^
	MeOH	2.33 ± 0.08 ^ab^	2.17 ± 0.28 ^abc^	52.53 ± 1.06 ^ab^	0.28 ± 0.01 ^ef^	0.07 ± 0.01 ^f^
	H_2_O	0.71 ± 0.06 ^g^	na	na	0.07 ± 0.01 ^g^	na
*S. ruthenica*	EA	2.21 ± 0.15 ^abc^	2.37 ± 0.11 ^a^	53.29 ± 1.10 ^a^	0.55 ± 0.01 ^a^	0.91 ± 0.01 ^bc^
	MeOH	2.29 ± 0.11 ^ab^	2.05 ± 0.05 ^abc^	55.59 ± 1.81 ^a^	0.27 ± 0.03 ^f^	0.10 ± 0.02 ^f^
	H_2_O	1.02 ± 0.09 ^f^	na	na	0.04 ± 0.01 ^g^	na
*S. stenoptera*	EA	2.41 ± 0.04 ^a^	2.01 ± 0.19 ^bc^	50.72 ± 8.32 ^ab^	0.50 ± 0.01 ^b^	0.79 ± 0.01 ^d^
	MeOH	1.89 ± 0.01 ^d^	1.21 ± 0.17 ^e^	54.40 ± 1.94 ^a^	0.35 ± 0.01 ^d^	0.08 ± 0.01 ^f^
	H_2_O	0.15 ± 0.03 ^h^	na	na	0.04 ± 0.01 ^g^	na

Values are reported as mean ± SD of three technical replications. GALAE: Galantamine equivalent; KAE: Kojic acid equivalent; ACAE: Acarbose equivalent; na: not active. Different letters in the same column indicate significant differences in the extracts (*p* < 0.05).

**Table 4:** Discriminant VIP compounds (VIP > 1.2) identified by AMOPLS modeling based on the effects of species, extraction solvent, and their interaction.

Species Factor		Extraction Factor		Species × Extraction	
Metabolite	VIP Score	Metabolite	VIP Score	Metabolite	VIP Score
p-Coumaroyl glycolic acid	2.8	Nonadecanoic acid	4.1	p-HPEA-EDA	2.1
Rhoifolin 4′-O-glucoside	2.8	4-Hydroxybenzaldehyde	4.1	Sinapine	2.1
Cirsimaritin	2.4	4-Vinylguaiacol	3.5	4-Hydroxybenzoic acid 4-O-glucoside	1.8
Eugenol	2.3	p-Coumaric acid	3.2	Syringic acid	1.7
Petunidin 3-O-(6″-p-coumaroyl-glucoside)	2.3	Isorhamnetin	3.2	5,6-Dihydroxy-7,8,3′,4′-tetramethoxyflavone	1.6
Hispidulin	2.0	4-Vinylphenol	3.0	3-Methoxynobiletin	1.6
Isorhamnetin 3-O-glucoside 7-O-rhamnoside	1.9	Dihydrosterculic acid	3.0	3-Hydroxyphloretin 2′-O-xylosyl-glucoside	1.6
Luteolin	1.9	Gallic acid	2.7	6-Geranylnaringenin	1.6
Biochanin A	1.8	(+)-Catechin	2.7	Gallic aldehyde	1.6
Apigenin 6,8-di-C-glucoside	1.7	Nobiletin	2.5	Phlorin	1.6
Chrysoeriol 7-O-(6″-malonyl-glucoside)	1.7	Thymol	2.3	Syringaresinol	1.6
Secoisolariciresinol	1.7	Kaempferol 3,7-O-diglucoside	2.2	Acetyl eugenol	1.6
Resveratrol	1.7	6-Prenylnaringenin	2.2	5-Heneicosylresorcinol	1.6
Spinacetin 3-O-glucosyl-(1-6)-[apiosyl(1-2)]-glucoside	1.7	Cyanidin 3,5-O-diglucoside	2.1	5-Tricosylresorcinol	1.6
Delphinidin 3-O-feruloyl-glucoside	1.7	Prodelphinidin dimer B3	2.1	24-Methylcholesterol ferulate	1.6
Luteolin 7-O-(2-apiosyl-glucoside)	1.7	p-Coumaric acid ethyl ester	2.0	Esculin	1.6
Sakuranetin	1.7	Carnosic acid	2.0	Schottenol ferulate	1.6
Pelargonidin 3-O-glucosyl-rutinoside	1.7	Methoxyphenylacetic acid	1.9	3,4-DHPEA-EDA	1.6
Rosmarinic acid	1.7	Isoxanthohumol	1.6	Phytanic acid	1.6
Diosmin	1.7	Sesamol	1.6	5-Pentacosenylresorcinol	1.6
6″-O-Acetylgenistin	1.7	Conidendrin	1.5	Sterculic acid	1.6
Patuletin 3-O-(2″-feruloylglucosyl)(1-6)-[apiosyl(1-2)]-glucoside	1.7	Petunidin 3-O-galactoside	1.5	Delphinidin 3-O-sambubioside	1.6
Quercetin 3-O-glucosyl-xyloside	1.7	3-p-Coumaroylquinic acid	1.4	7,3′,4′-Trihydroxyflavone	1.6
Phloretin	1.7	Rosmadial	1.4	Arctigenin	1.6
Quercetin 3-O-galactoside	1.7	Cinnamic acid	1.4	Kaempferol 3,7,4′-O-triglucoside	1.6
Cyanidin 3-O-(6″-p-coumaroyl-glucoside)	1.7	Ferulic acid 4-O-glucoside	1.4	Naringin	1.6
Matairesinol	1.7	Dimethylmatairesinol	1.4	Delphinidin 3-O-galactoside	1.6
3-Methylcatechol	1.6	3-Feruloylquinic acid	1.4	Cyanidin 3-O-glucosyl-rutinoside	1.6
Tetramethylscutellarein	1.6	Caffeoyl aspartic acid	1.3	6″-O-Acetyldaidzin	1.6
Tyrosol	1.5	Piceatannol	1.2	[6]-Gingerol	1.6
Hydroxytyrosol 4-O-glucoside	1.4			Eriodictyol	1.5
Luteolin 6-C-glucoside	1.4			Secoisolariciresinol-sesquilignan	1.5
4-Ethylphenol	1.4			5-Heneicosenylresorcinol	1.5
Medioresinol	1.4			Vanillic acid	1.4
Demethoxycurcumin	1.4			Piceatannol 3-O-glucoside	1.2
Cyanidin 3-O-rutinoside	1.4			1,4-Naphtoquinone	1.2
d-Viniferin	1.4				
Chrysoeriol 7-O-glucoside	1.3				
(+)-Catechin 3-O-glucose	1.3				
3,4-Dihydroxyphenylglycol	1.3				
Sinapaldehyde	1.2				
Kaempferol 3-O-glucosyl-rhamnosyl-galactoside	1.2				
Coumarin	1.2				

**Table 5:** Semi-quantitative analysis of various classes of phenolic compounds in five Salsola species extracted with EA, MeOH, and H_2_O.

Species	Extraction	Anthocyanins (µg CyE g^−1^ FW)	Flavanols (µg CaE g^−1^ FW)	Flavones (µg LE g^−1^ FW)	Flavanols (µg QE g^−1^ FW)	Lignans (µg SE g^−1^ FW)	LMW and Other Polyphenols (µg TE g^−1^ FW)	Ph.Acids (µg FE g^−1^ FW)
*S. crassa*	EA	0 ± 0 b	0 ± 0 c	19.0 ± 0.4 d	9.8 ± 0.7 c	1172.2 ± 19.2 a	462.1 ± 44.6 a	130.7 ± 3.5 d
*S. kali*		43.8 ± 4.1 a	4.8 ± 0.5 b	115.1 ± 5.0 a	327.0 ± 10.2 a	1054.6 ± 16.4 c	310.9 ± 17.3 b	113.5 ± 13.0 d
*S. nitraria*		0 ± 0 b	0.0 ± 0 c	32.2 ± 0.8 b	42.0 ± 1.5 b	1126.3 ± 27.1 b	362.0 ± 31.5 b	262.8 ± 16.9 c
*S. ruthenica*		0 ± 0 b	264.5 ± 2.3 a	25.4 ± 2.8 c	17.2 ± 8.1 c	20.3 ± 1.2 e	309.9 ± 72.9 b	787.0 ± 44.6 a
*S. stenoptera*		0 ± 0 b	0 ± 0 c	8.7 ± 1.6 e	17.1 ± 4.6 c	464.9 ± 13.6 d	77.3 ± 3.1 c	641.3 ± 110.3 b
*S. crassa*	MeOH	153.5 ± 4.3 a	224.9 ± 2.5 b	62.2 ± 1.2 c	676.7 ± 10.4 a	0 ± 0 c	549.1 ± 49.8 c	627.5 ± 35.8 a
*S. kali*		105.9 ± 5.2 b	34.6 ± 2,7 e	206.9 ± 9.3 a	500.0 ± 21.4 b	43.8 ± 12.1 b	825.8 ± 45.7 c	169.0 ± 17.8 b
*S. nitraria*		11.9 ± 1.2 d	195.7 ± 7.2 c	18.2 ± 3.0 d	85.3 ± 6.0 e	5.2 ± 4.8 c	1575.5 ± 20.1 b	33.8 ± 1.8 c
*S. ruthenica*		57.0 ± 1.2 c	103.4 ± 1.2 d	226.4 ± 22.1 a	329.2 ± 4.9 c	821.9 ± 38.4 a	5837.1 ± 336.3 a	30.3 ± 0.6 c
*S. stenoptera*		16.4 ± 0.6 d	233.7 ± 6.8 a	91.8 ± 4.2 b	149.9 ± 4.8 d	28.8 ± 3.9 bc	1438.9 ± 24.8 b	9.0 ± 2.1 c
*S. crassa*	H_2_O	17.8 ± 1.9 a	78.1 ± 4.1 b	10.2 ± 0.6 c	102.3 ± 5.5 a	10.8 ± 0.9 d	299.4 ± 39.2 c	10.8 ± 2.4 e
*S. kali*		6.7 ± 0.5 c	19.6 ± 0.8 d	32.7 ± 2.3 b	42.3 ± 3.0 c	73.3 ± 3.2 b	581.4 ± 99.6 d	208.1 ± 6.5 b
*S. nitraria*		0.0 ± 0 e	197.0 ± 1.7 a	10.3 ± 1.9 c	19.1 ± 1.1 d	6.9 ± 1.0 d	10,508.1 ± 70.1 a	230.1 ± 32.2 a
*S. ruthenica*		9.9 ± 1.1 b	56.7 ± 3.1 c	42.5 ± 3.5 a	62.8 ± 6.6 b	347.1 ± 12.1 a	7114.9 ± 167.7 b	49.0 ± 4.4 d
*S. stenoptera*		2.1 ± 0.1 d	198.0 ± 5.0 a	30.0 ± 0.7 b	49.5 ± 1.7 c	33.7 ± 3.3 c	488.7 ± 51.1 c	145.8 ± 8.0 c

The data represent the average of three technical replicates. The different letters indicate statistical differences. The data were from the analysis of variance (ANOVA) and Duncan’s post hoc test, with a significance level of *p* < 0.05.

## Data Availability

The original contributions presented in this study are included in the article/Supplementary Material. Further inquiries can be directed to the corresponding author.

## Supplementary Materials

The following supporting information can be downloaded at: https://www.mdpi.com/article/10.3390/plants15020199/s1. Table S1: Metabolomics dataset obtained from untargeted UHPLC-ESI-QTOF-MS; Table S2: Statistical analysis from AMOPLS analysis for Species, Extraction, and Species x Extraction interaction; Table S3: Discriminating VIP markers obtained from AMOPLS analysis; Table S4: Multivariate analysis of variance (MANOVA) assessing the effects of species, extraction solvent, and their interaction on phenolic composition; Table S5: Semi-quantitative analysis of various classes of phenolic compounds in five Salsola species extracted with EA, MeOH, and H2O; Table S6: Correlation and p-value tables from the Pearson heatmap analysis; Table S7: Location of the plant samples; Figure S1: Total and factor-specific VIP^2^ (Variable Importance in Projection) scores for the 50 compounds contrib-uting most significantly to Species, Extraction, and their interaction effects. Metabolite names corresponding to these compounds are provided in Supplementary Table S3.
