# Supplementary material for: The Metabolic Diversity of Different Salsola Species Valorized Through Untargeted Metabolomics and In Vitro Bioassays: The Importance of Phenolic Constituents
Source: Plants (Basel). 2026 Jan 8;15(2):199. doi: 10.3390/plants15020199 (PMC12844799; doi:10.3390/plants15020199)
Supplement: Supplementary file 1 [file plants-15-00199-s001.zip › Supplemental materials_PLANTS.pdf]

The metabolic diversity of different *Salsola* species valorized through untargeted metabolomics and *in vitro* bioassays: the importance of phenolic constituents

Hajar Salehi[1], Marco Armando De Gregorio[1], Gokhan Zengin[2], Sakina Yagi[3], Gunes Ak[2], Enver Saka[2], Fevzi Elbasan[4], Evren Yıldıztugay[4], Leilei Zhang[1], Stefano Dall'Acqua[5*], Luigi Lucini[1]

1*Department for Sustainable Food Process, Università Cattolica del Sacro Cuore, Piacenza, Italy,* hajar.salehi@unicatt.it (HS), marcoarmando.degregorio@unicatt.it (MD); leilei.zhang@unicatt.it (LZ), luigi.lucini@unicatt.it (LL)
2*Department of Biology, Science Faculty, Selcuk University, Konya, Turkey ,* gokhanzenginselcuk.edu.tr (GZ), akguneselcuk@gmail.com (GA), enverss2016@gmail.com (ES)
3*Department of Botany, Faculty of Science, University of Khartoum, Khartoum, Sudan.* sakinayagi@gmail.com (SY)
4*Department of Biotechnology, Science Faculty, Selcuk University, Konya, Turkey. fevzi.elba@gmail.com (FE); eytugay@gmail.com (EY)*
5*Department of Pharmaceutical and Pharmacological Sciences, University of Padova, Via Marzolo 5, 35131 Padova, Italy*
* Correspondence: stefano.dallacqua@unipd.it

Table of Contents

Table S4. Multivariate analysis of variance (MANOVA) assessing the effects of species, extraction solvent, and their interaction on phenolic composition……………………………………………….2

Table S5. Semi-quantitative analysis of various classes of phenolic compounds in five *Salsola* species extracted with EA, MeOH, and H2O. ………………………………………………………………..3

Table S7 Location of the plant samples…………………………………………………………………3

Figure S1. Total and factor-specific VIP² (Variable Importance in Projection) scores for the 50 compounds contributing most significantly to Species, Extraction, and their interaction effects. Metabolite names corresponding to these compounds are provided in Supplementary Table 3S..…4

Table S4. Multivariate analysis of variance (MANOVA) assessing the effects of species, extraction solvent, and their interaction on phenolic composition.

Effect	Test Statistic	Value	F-value	Hypothesis df	Error df	p-value
Intercept	Wilks' Lambda	0.000	6537.389	8	23	<0.001
Species	Wilks' Lambda	0.000	1407.070	32	86.415	<0.001
Extraction	Wilks' Lambda	0.000	5139.092	16	46	<0.001
Species × Extraction	Wilks' Lambda	0.000	943.782	64	139.152	<0.001

Table S5. Semi-quantitative analysis of various classes of phenolic compounds in five *Salsola* species extracted with EA, MeOH, and H_2O.

Variables	Anthocyanins (μg CyE g^{-1} FW)	Flavanols Flavanols (μg CaE g^{-1} FW)	Flavones (μg LE g^{-1} FW)	Favonols Favonols (μg QE g^{-1} FW)	Lignans (μg SE g^{-1} FW)	LMW and other polyphenols (μg TE g^{-1} FW)	Ph.Acids (μg FE g^{-1} FW)
S. crassa	57.1 ± 2.1 a	101.0 ± 2.2 c	30.5 ± 0.7 d	262.9 ± 5.5 b	394.3 ± 6.7 ab	463.9 ± 44.5 d	253.3 ± 13.9 a
S. kali	52.1 ± 3.3 b	19.7 ± 1.4 d	118.2 ± 5.5 a	289.8 ± 11.5 a	390.5 ± 10.6 ab	572.7 ± 54.2 c	163.5 ± 12.4 b
S. Nitraria	4.0 ± 0.4 e	130.9 ± 3.0 b	20.2 ± 1.9 e	48.8± 2.9 e	379.4 ± 11.0 b	4148.5 ± 40.6 b	175.6 ± 14.0 b
S. ruthenica	22.3 ± 0.8 c	141.6 ± 2.2 a	98.1 ± 9.5 b	136.4 ± 6.5 c	396.4 ± 17.2 a	4420.7 ± 191.6 a	288.8 ± 16.5 a
S.Stenoptera	6.2 ± 0.2 d	143.9 ± 3.9 a	43.5 ± 2.2 c	72.2 ± 3.7 d	175.8 ± 6.9 c	668.3 ± 26.3 c	265.3 ± 40.2 a
Sig	***	***	***	***	***	***	***
EA	8.8 ± 0.8 b	53.9 ± 0.6 b	40.1 ± 2.1 b	82.6 ± 5.0 b	767.7 ± 15.5 a	304.4 ± 33.9 c	387.1 ± 37.6 a
MeOH	69.0 ± 2.5 a	158.5 ± 4.1 a	121.1 ± 8.0 a	348.2 ± 9.5 a	179.9 ± 11.9 b	2045.3 ± 95.3 b	173.9 ± 11.6 b
H_2O	7.3 ± 0.7 b	109.9 ± 2.9 b	25.1 ± 1.8 c	55.2 ± 3.6 c	94.4 ± 4.1 c	3798.5 ± 85.1 a	128.8 ± 8.9 c
Sig	***	***	***	***	***	***	***

The different letters indicate statistical differences. The data were from a two-way analysis of variance (MANOVA) followed by Duncan's post hoc test at a significance level of $p < 0.05$. *** represents a significant level of $p < 0.0001$.

Table S7. Location of the plant samples

***Salsola* species**	**Location**
Salsola kali L	Anamur/Mersin, Pullu Camp Area, Voucher No: EY-3069
Salsola crassa Bieb	Cihanbeyli/Konya, around Tersakan Lake, Voucher No: EY-3084
Salsola nitraria Pallas	Şereflikoçhisar/Ankara, around Koyuncu Salt Factory, Voucher No: EY-3085
Salsola stenoptera Wagenitz	Cihanbeyli/Konya, Yavşan Location, Voucher No: EY-3095
Salsola ruthenica Iljin	Selçuklu/Konya, Yazır Location, Voucher No: EY-3135

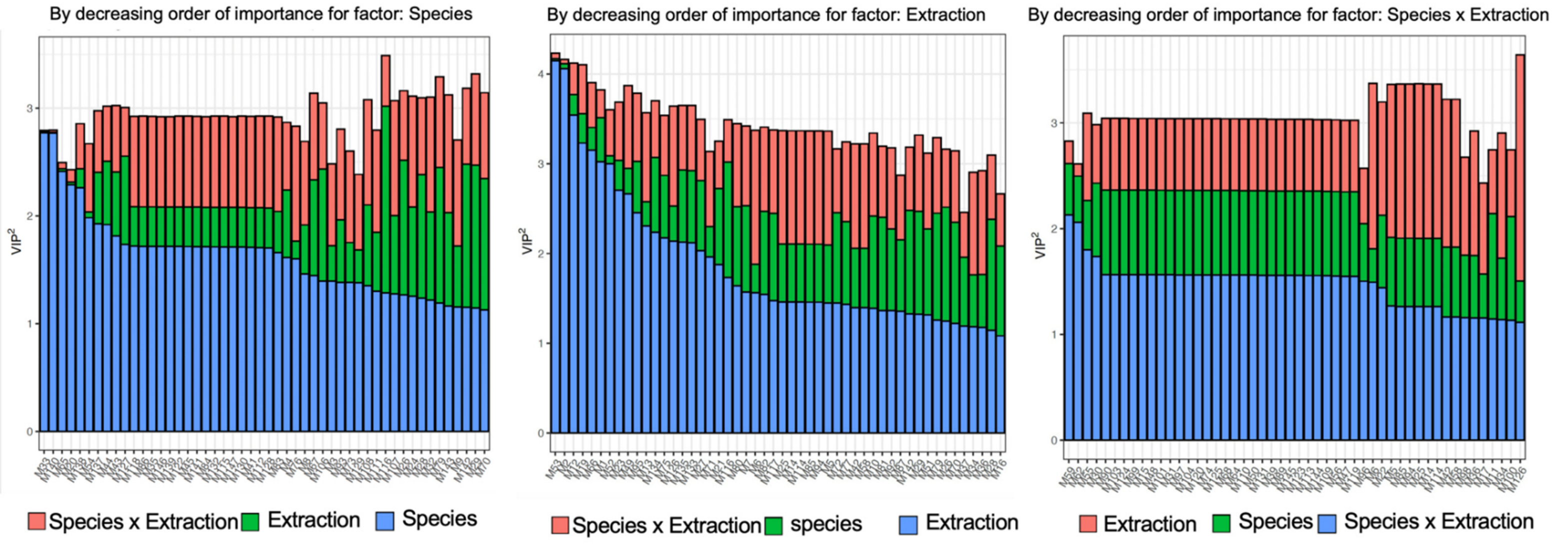

Figure S1. Total and factor-specific VIP² (Variable Importance in Projection) scores for the 50 compounds contributing most significantly to Species, Extraction, and their interaction effects. Metabolite names corresponding to these compounds are provided in Supplementary Table 3S.
